# Local and abscopal responses in advanced intrahepatic cholangiocarcinoma with low TMB, MSS, pMMR and negative PD-L1 expression following combined therapy of SBRT with PD-1 blockade

**DOI:** 10.1186/s40425-019-0692-z

**Published:** 2019-08-05

**Authors:** Xiaoliang Liu, Jianfei Yao, Lele Song, Sujing Zhang, Tanxiao Huang, Yu Li

**Affiliations:** 1 Department of Radiotherapy, the Chinese PLA General Hospital, No.28, Fuxing Road, Haidian District, Beijing, 100039 People’s Republic of China; 2 HaploX Biotechnology, Co., Ltd., 8th floor, Auto Electric Power Building, Songpingshan Road, Nanshan District, Shenzhen, 518057 Guangdong People’s Republic of China; 3 Department of Radiotherapy, the eighth medical center of the Chinese PLA General Hospital, No.17, Heishanhu Road, Haidian District, Beijing, 100091 People’s Republic of China; 4 The First Hospital of Hebei Medical University, No.89, Donggang Road, Yuhua District, Shijiazhuang, 050051 Hebei Province People’s Republic of China

**Keywords:** Intrahepatic cholangiocarcinoma, Radiotherapy, Immunotherapy, PD-1, PD-L1, TMB, MMR, MSI, Abscopal response

## Abstract

**Background:**

Late-stage or recurrent intrahepatic cholangiocarcinoma (ICC) patients exhibit poor prognosis due to limited sensitivity to chemotherapy or radiotherapy and coexistence of multiple lesions. Programmed cell death protein 1 (PD-1) blockade provides a therapeutic opportunity for patients with high tumor mutation burden (TMB), high microsatellite instability (MSI-H), deficient mismatch repair (dMMR) and/or positive programmed cell death ligand 1 (PD-L1) expression. However, it is currently believed that patients with low TMB, microsatellite stable (MSS), proficient mismatch repair (pMMR) or negative PD-L1 expression are less likely to benefit from PD-1 blockade.

**Case presentation:**

Here we provide the first report on the therapeutic responses of ICC patients treated with combined PD-1 blockade with stereotactic body radiotherapy (SBRT) (Cyberknife) in the background of low TMB, MSS, pMMR and negative PD-L1 expression. One stage IVA ICC patients and two postsurgical recurrent ICC patients were involved in this study and the responses of both locally irradiated tumor(s) and the abscopal tumors or metastasis to the combined therapy were assessed by magnetic resonance imaging (MRI) and positron emission tomography-computed tomography (PET-CT). The stage IVA ICC patient (patient A) exhibited a TMB of 1.2 muts/Mb with MSS, pMMR and < 1% PD-L1 expression. Both the intrahepatic lesion and the lymph node metastases were well controlled for 7 months, and partial response (PR) was achieved with the sum of lesion diameters decreased by 40.9%. One of the postsurgical recurrent ICC patients (Patient B) exhibited a TMB of 3.8 muts/Mb with MSS, pMMR and < 1% PD-L1 expression. Both the recurrent intrahepatic lesion and the lymph node metastases were well controlled by the combined therapy and the sum of lesion diameter decreased by 86.3% (PR). The other postsurgical recurrent patient (Patient C) exhibited a TMB of 0.98 muts/Mb with MSS, pMMR and < 1% PD-L1 expression, and achieved complete response (CR) and maintained for 11 months. Abscopal effects were observed for all three patients.

**Conclusions:**

This study provided the first set of evidence for the effectiveness of SBRT and PD-1 blockade combined therapy in late-stage or recurrent ICC patients with low TMB, MSS, pMMR and negative PD-L1 expression, and potentially expanded the indications of the combined therapy to those patients who were previously not suitable for immunotherapy.

## Introduction

Cholangiocarcinoma is classified into intrahepatic cholangiocarcinoma (ICC) and extrahepatic cholangiocarcinoma. The incidence of ICC in the US increased from 0.49 per 100,000 in 1995 to 1.49 per 100,000 in 2014, with an average annual increase rate of 5.49% [1]. In contrast, the incidence of ICC in China was reported to be approximately 6 per 100,000 [2, 3] and the mortality rate was about 1.86 per 100,000 [4]. Surgery is regarded as the only primary curative treatment for ICC. Nevertheless, more than two-thirds of patients are inappropriate for surgery at diagnosis, and more than 60% of patients relapse after surgery [5]. Therefore, the prognosis of advanced ICC is poor, and the five-year survival following resection ranges from 14 to 40% [6]. Surgery is not recommended for unresectable or metastatic ICC, while palliative therapy, such as transcatheter arterial chemoembolization (TACE), radiofrequency ablation (RFA), radiotherapy and chemotherapy are recommended. Gemcitabine+cisplatin (GC) and Gemcitabine+SI (GS) are widely used as the standard chemotherapy for unresectable or metastatic ICC. Meanwhile, GC and GS plans are also standard therapies for post-surgical ICC patients [7, 8]. Several prognostic factors, including curative resection (R0), the number of tumors (single or multiple), and the presence of vascular invasion and lymph node metastases are suggested as the most important independent predictors of survival [9].

Current therapeutic options for advanced or recurrent ICC are limited. Conventional chemotherapy, radiotherapy (RT) or radiochemotherapy do not show satisfactory responses [10–12]. In recent years, immunotherapy targeting PD-1/PD-L1 has achieved encouraging therapeutic effects in diverse cancers, and NCCN guidelines recommend pembrolizumab as a choice for advanced cholangiocarcinoma with dMMR or MSI-H [13–16]. Immunotherapy combined with targeted therapy or chemotherapy in ICC treatment has recently been investigated and exhibited promising therapeutic perspectives, although more evidence is still needed to confirm the efficacy [16–19]. Immunotherapy combined with radiotherapy, on the other hand, might be a potential alternative therapy for ICC [20–22]. However, no studies have been performed to investigate the therapeutic effectiveness of the combination in ICC, although it has shown promising therapeutic responses in melanoma, non-small cell lung cancer (NSCLC), neuroendocrine cervical carcinoma and refractory Hodgkin’s lymphoma [23–30]. It appears from these reports that radiotherapy administered before, after or concurrently with immunotherapy, all exhibited promising therapeutic response. The rationale behind the combined effect of radiotherapy with immunotherapy has been investigated. It was suggested that the effects of radiation in sensitizing the immunotherapy may result from the modification of the tumor microenvironment that can interfere with tumor resistance to immunotherapy. Ionizing radiation may enable the generation of a tumor-specific immune response. This includes a series biological process, including angiogenesis, vasculogenesis and fibroblasts, etc., mediated by a variety of inflammatory cells [31].

In the present report, we performed the first study investigating the responses of late-stage or recurrent ICC to the combined therapy of PD-1 blockade with SBRT in patients with low TMB, MSS, pMMR and negative PD-L1 expression. We found that the combination achieved satisfactory responses in ICC patients, which may expand its applications to patients who were previously regarded as not suitable for immunotherapy.

## Case presentations

### Patient A

**Fig. 1:**
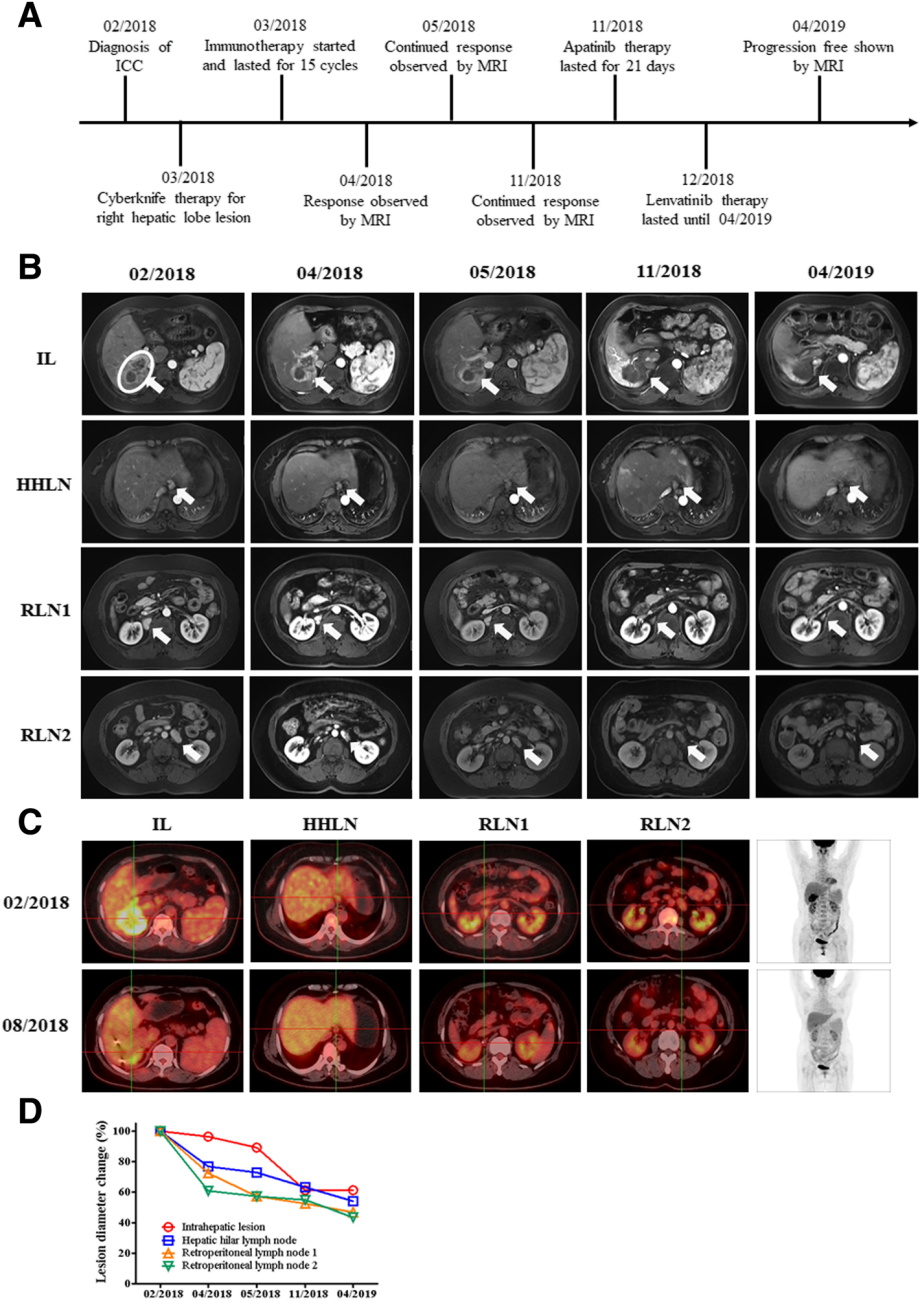
Images describing the condition and therapeutic responses of patient A. **a** scheme shows the time course of patient A in diagnosis the therapy. **b** MRI images show the therapeutic response of patients following a series of treatment. The circle indicates the target lesion/region for radiotherapy, and arrows in figures indicate the position of lesions. **c** PET-CT images show the therapeutic response of patients following a series of treatment. **d** Statistics of lesion diameter reduction for all lesions of patient A. IL: Intrahepatic lesion, HHLN: Hepatic hilar lymph node, RLN1: Retroperitoneal lymph node 1, RLN2: Retroperitoneal lymph node 2

A 52-year-old female patient with 27-year history of positive hepatitis B surface antigen (HBsAg) was diagnosed with stage IVA ICC. Abdominal MRI revealed a solid mass in the right liver lobe with lymph node metastases in hepatic hilar and retroperitoneum (Fig. 1a). Whole-exome sequencing (WES) on needle biopsy sample of the intrahepatic lesion before therapy revealed TMB of 1.2 mut/Mb with pMMR and MSS, and immunohistochemistry revealed PD-L1 expression level of < 1%. She underwent SBRT (Cyberknife) therapy for the right hepatic lobe lesions with 55Gy/5F, and received immunotherapy with nivolumab at a dose of 200 mg every 2 weeks for 15 cycles. One month after the initiation of the combined therapy, MRI revealed remarkable necrosis of the locally irradiated intrahepatic lesion, and volume reduction of the abscopal nonirradiated lymph node metastases in hepatic hilar and retroperitoneum were also observed (Fig. 1b, d). MRI revealed that both the irradiated and nonirradiated lesions continued to shrink and remained stable from 2 months to 8 months after the initiation of the combined therapy (Fig. 1b, d). Although the lesions can still be observed by MRI, the PET-CT showed decreased metabolic activities in the intrahepatic lesions and disappeared hypermetabolic activities in lymph nodes of hepatic hilar and retroperitoneum 5 months after the initiation of the combined therapy (Fig. 1b, c, d). Subsequent maintenance therapy was implemented with apatinib and lenvatinib (Fig. 1a & b). With the combination of SBRT and nivolumab, the diameter of intrahepatic irradiated lesions decreased by 38.9%, and the diameters of nonirradiated lesions by 36.7%~ 47.8% after 13 months of the initiation of combined therapy, achieving overall PR with the sum of the diameter decreased by 40.9%. No obvious toxicity associated with the combined therapy was observed (Fig. 1b, c, d).

### Patient B

**Fig. 2:**
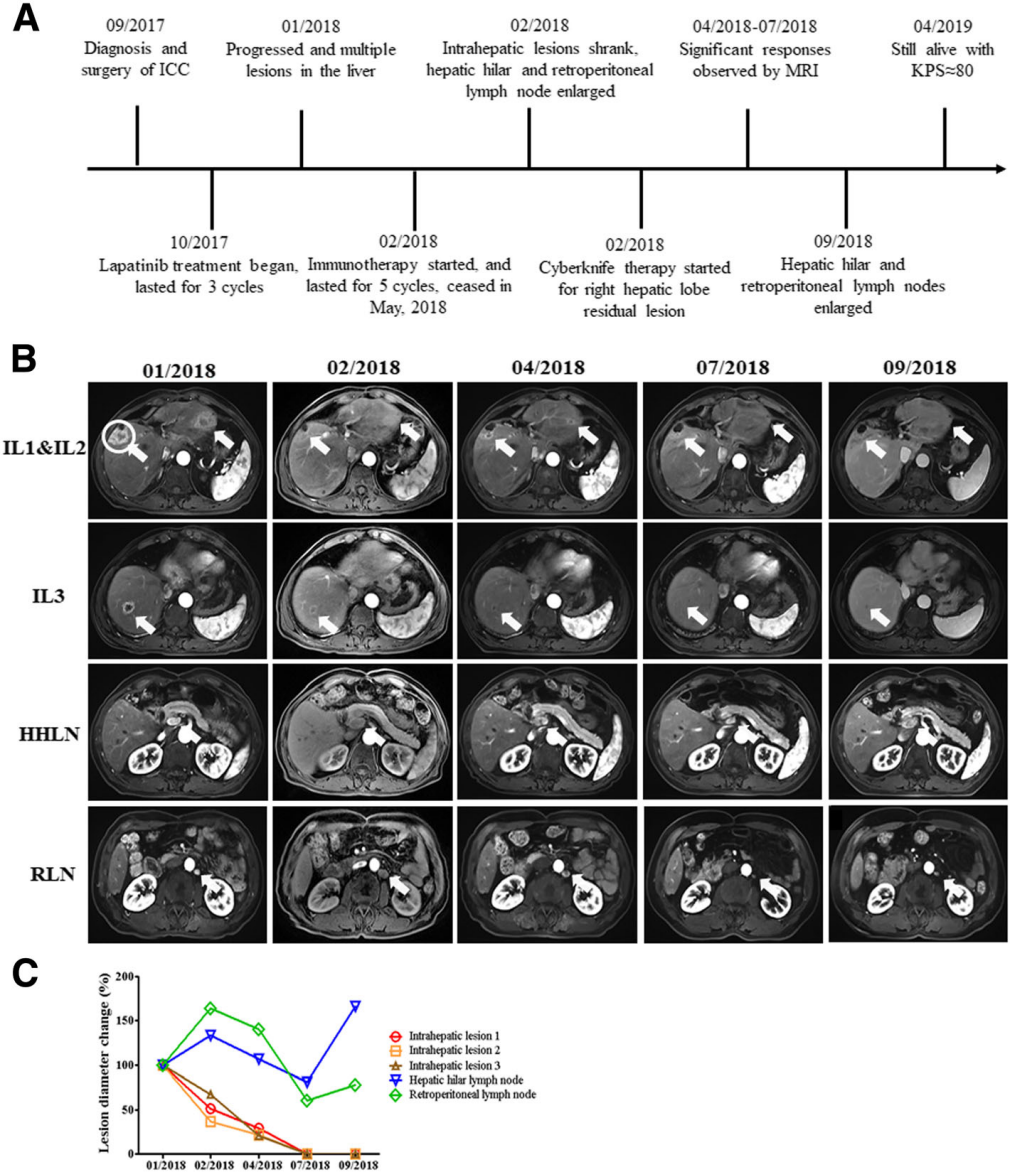
Images describing the condition and therapeutic responses of patient B. **a** scheme shows the time course of patient B in diagnosis the therapy. **b** MRI images show the therapeutic response of patients following a series of treatment. The circle indicates the target lesion/region for radiotherapy, and arrows in figures indicate the position of lesions. **c** Statistics of lesion diameter reduction for all lesions of patient B. Arrows in figures indicate the position of lesions. IL1: Intrahepatic lesion 1, IL2: Intrahepatic lesion 2, IL3: Intrahepatic lesion 3, HHLN: Hepatic hilar lymph node, RLN: Retroperitoneal lymph node

A 59-year-old male patient with HBV infection history for more than 30 years underwent surgical resection of the middle hepatic lobe, and was diagnosed with stage IIIA ICC (Fig. 2a). WES was performed with primary intrahepatic tumor and showed a TMB of 3.8 muts/Mb with MSS and pMMR, and immunohistochemistry showed PD-L1 expression of < 1%. The amplification of ERBB2 was detected, and lapatinib was used after surgery as a kinase inhibitor of ERBB2. Post-surgical lapatinib treatment for 3 cycles did not control the tumor growth well. The primary tumor relapsed and multiple new lesions at both left and right lobes appeared with hepatic hilar and retroperitoneal lymph node metastases 3 months after the start of lapatinib therapy (Fig. 2b, c). Immunotherapy with pembrolizumab then started and continued for 5 cycles, and MRI showed decreased intrahepatic lesions but enlarged hepatic hilar and retroperitoneal lymph node metastases after 1 cycle of immunotherapy (Fig. 2b, c). Subsequent Cyberknife therapy targeting the right hepatic lobe lesion was implemented with 52Gy/4F. MRI showed significant response to the combined SBRT with Pembrolizumab therapy from 2 to 5 months after the start of the combined therapy (Fig. 2b, c). The immunotherapy stopped after 5 cycles due to personal reasons of the patient. All intrahepatic lesions continued to shrink after the stop of immunotherapy, with the diameter of intrahepatic irradiated lesions decreased by 100% (CR). In contrast, hepatic hilar and retroperitoneal lymph nodes continued to shrink first for 5 months but enlarged again thereafter, with the diameters decreased by 18.7%~ 100% (PR) by 2 months after the end of immunotherapy (Fig. 2b, c). The patient achieved overall PR with the sum of the lesion diameters decreased by 86.3%, and no obvious toxicity associated with the combined therapy was observed.

### Patient C

**Fig. 3:**
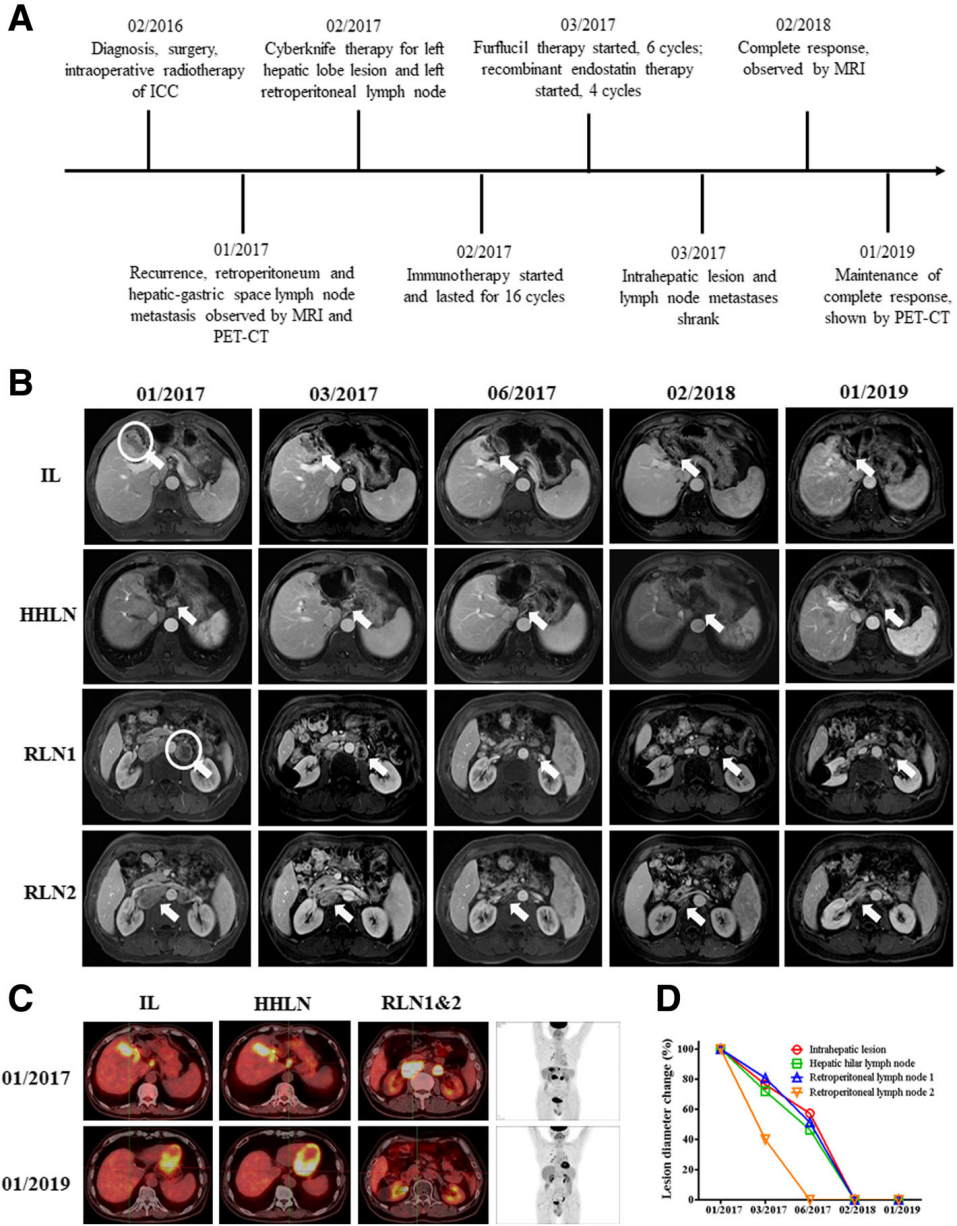
Images describing the condition and therapeutic responses of patient C. **a** scheme shows the time course of patient A in diagnosis the therapy. **b** MRI images show the therapeutic response of patients following a series of treatment. The circles indicate the target lesions/regions for radiotherapy, and arrows in figures indicate the position of lesions. **c** PET-CT images show the therapeutic response of patients following a series of treatment. **d** Statistics of lesion diameter reduction for all lesions of patient C. Arrows in figures indicate the position of lesions. IL: Intrahepatic lesion, HHLN: Hepatic hilar lymph node, RLN1: Retroperitoneal lymph node1, RLN2: Retroperitoneal lymph node2

A 51-year-old male patient with ten-year HBV infection history underwent surgical resection of the lesion in left hepatic lobe, and was diagnosed with stage IIIB ICC. MRI and PET-CT revealed the right lobe ICC recurrence with hepatic hilar and retroperitoneal lymph node metastasis 11 months after the surgery. WES revealed a TMB of 0.98 muts/Mb with pMMR and MSS, and immunohistochemistry revealed PD-L1 expression level of < 1%. He underwent Cyberknife therapy for left hepatic lobe lesion and left retroperitoneal lymph node with 52Gy/4F. Subsequent immunotherapy with pembrolizumab at a dose of 200 mg every 3 weeks lasted for 16 cycles, and chemotherapy with Furflucil (1-(2-Tetrahydrofuryl)-5-fluorouracil) lasted for 6 cycles, and recombinant human endostatin lasted for 4 cycles (Fig. 3a). One month after the initiation of the combined therapy, MRI revealed that the intrahepatic irradiated and nonirradiated lymph node lesions all shrank (Fig. 3b, d), and continued to shrink for 4 months (Fig. 3b, d). Subsequent monitoring by MRI and PET-CT revealed that the patient achieved complete response (CR) 12 months after the initiation of the combined therapy (Fig. 3b, c, d). He maintained CR for 11 months till January, 2019 (Fig. 3b, c, d). No obvious toxicity associated with the combined therapy of SBRT with PD-1 blockade was observed.

## Discussion

Advanced ICCs have a poor prognosis due to the low resection rate and high relapse rate. It is imperative to explore new effective treatment strategy of ICC. The newly updated NCCN guidelines recommended PD-1 blockade for ICC patients with dMMR or MSI-H. The recommendation was based on a series studies showing that cholangiocarcinoma patients can benefit from immunotherapy. One initial study including 4 patients with cholangiocarcinoma showed the efficacy of PD-1 blockade for dMMR cancers, in which one patient showed CR and others had stable disease, resulting in a disease control rate (DCR) of 100% [16]. Another study investigated the efficacy of nivolumab in 29 patients with advanced refractory biliary tract cancers. The DCR reached 55% as 5 patients achieved PR and 11 had stable disease (SD) [17]. The median PFS was 3.5 months (95% CI: 2.1–7.6) and the median OS had not been reached, and the 6-month OS was 76.3% for all 34 patients with median follow-up of 8 months [17]. The combination of immunotherapy (pembrolizumab) with targeted therapy (ramucirumab) was shown to be effective for advanced cholangiocarcinoma, and patients with PD-L1 positive expression exhibited improved overall survival compared with PD-L1 negative patients [18]. Furthermore, the combination of lenvatinib with pembrolizumab or nivolumab achieved an overall response rate (ORR) of 21.4% and DCR of 92.9% in 14 Stage IV ICC patients who had more than two lines of anticancer therapy, and high TMB was strongly associated with a better therapeutic response [19]. Combined therapy of PD-1 blockade with chemotherapy emerged very recently as a new option for advanced or recurrent ICC, and a few case reports showed promising results: patients with high TMB or high INDEL mutation frequency achieved marked response to the combined therapy [32, 33]. It appeared that ICC patients with high TMB, MSI-H, dMMR and/or PD-L1 positive expression can benefit from immunotherapy or its combination with targeted therapy or chemotherapy.

The combination of immunotherapy with radiotherapy has been shown to be an effective therapy in a few cancers (Table 1). The first study of the combined therapy reported a melanoma case in 2012, showing that the combination of ipilimumab with radiotherapy induced abscopal effect, which relieved both irradiated lesions and nonirradiated lesions [24]. Subsequent studies in melanoma expanded the sample size and investigated the performance of radiotherapy combined with nivolumab, pembrolizumab or ipilimumab [25–27]. One retrospective analysis compared the treatment response of combined radiotherapy/ipilimumab to that of ipilimumab alone in 101 patients with melanoma (Table 1). The median overall survival and the rate of complete response were significantly higher in the combined therapy than ipilimumab alone [25] (Table 1). There are many studies that investigated the efficacy of immunotherapy and radiotherapy combination in NSCLC. One meta-analysis focusing on metastatic NSCLC included 18 studies and concluded that the combination had a good safety profile and achieved high rates of local control and greater chances of obtaining abscopal responses than radiotherapy alone, with a relevant impact on PFS [28] (Table 1). The efficacy of the combination has also been studied in neuroendocrine cervical carcinoma and refractory Hodgkin’s lymphoma [29, 30]. Furthermore, one recent study reported the efficacy of the combined therapy of SBRT with pembrolizumab in 79 solid tumor patients covering 27 cancer types [34]. Multisite SBRT followed by pembrolizumab was well tolerated with acceptable toxicity, and achieved an overall ORR of 13.2% with median OS of 9.6 months, median PFS of 3.1 months and nonirradiated ORR of 26.9% (Table 1). Studies reviewed in Table 1 indicate that the combined radiotherapy with immunotherapy exhibited good therapeutic efficacy with low toxicity in a majority of cancer types.

**Table 1:** Summary of representative studies on the combination of radiotherapy with immunotherapy in main cancer types investigated to date

Cancer type	Patient Characteristics	Number of patients	Status of TMB/MSI/MMR/PD-L1	Treatment	Outcome	References
Melanoma	advanced melanoma	25	Not Specified	RT + nivolumab or pembrolizumab	CR&PR&SD&PD in irradiated lesions: 24,12,24,32%;in nonirradiated lesions: 20, 19, 12, 40%	[25]
	metastatic melanoma	59	Not Specified	17:RT + nivolumab or pembrolizumab 42:nivolumab or pembrolizumab	RT + anti-PD-1 therapy vs anti-PD-1 therapy, ORR: 64.7% VS 33.3%;	[26]
	metastatic melanoma	101	Not Specified	70: concurrent radiotherapy with ipilimumab (Ipi-RT); 31 ipilimumab alone	OS significantly increased (19 vs 10 months). Median PFS marginally increased (5 vs 3 months). CR rate significantly increased (25.7% vs 6.5%), OR rate increaed (37.1% vs 19.4%).	[24]
NSCLC	recurrence after at least 1 prior platinum-containing regimen	1736 (18 studies)	Not Specified	RT + nivolumab, or pembrolizumab, or ipilimumab	Local Control Rate (CR + PR + S): 70.7%; median OS: 12.4 months; PFS: 4.6 months;Distant/Abscopal Response Rate (CR + PR + S):41.3%;Toxicity ≥Grade 3:20.0%	[27]
Solid tumors	metastatic solid tumor previously treated with standard-of-care therapy	73 (27 cancer types)	Not Specified	RT + pembrolizumab	overall ORR: 13.2%; Median OS: 9.6 months; Median PFS: 3.1 months; nonirradiated ORR: 26.9%	[34]

*TMB* tumor mutation burden, *MSI* microsatellite instability, *MMR* mismatch repair, *PD-L1* programmed death ligand-1, *NSCLC* non-small cell lung cancer, *CR* complete response, *PR* partial response, *SD* stable disease, *PD* progressive disease, *OS* overall survival, *ORR* objective response rate, *PFS* progression free survival, *RT* radiotherapy

Studies on the combination of immunotherapy with chemotherapy or targeted therapy showed that ICC patients with high TMB, dMMR, MSI or positive PD-L1 expression exhibited better response, while most studies on the combination of immunotherapy with radiotherapy in various cancers did not describe the status of TMB, MMR, MSS or PD-L1 expression. Our present study showed that late-stage or recurrent ICC patients can also benefit from the combination of immunotherapy with SBRT, even if they had low TMB, pMMR, MSS or negative PD-L1 expression. The combined therapy appeared to be effective regardless of the sequence of immunotherapy or SBRT. This suggests a huge potential advantage of immunotherapy combined with SBRT, since there are many cancer patients with low TMB, pMMR, MSS or negative PD-L1 expression, not only in ICC, but also in other cancers. This combination provides a new effective option for their therapy.

Strong abscopal effects were observed in all three patients in this study. Both irradiated and nonirradiated lesions responsed to the combination of radiotherapy and immunotherapy, and the responses in nonirradiated lesions, such as the lymph node metastases in patient A and C, were even better than the primary lesions. These observations suggest that the responses in these patients may be due to the combination of radiotherapy and immunotherapy. Since advanced ICC had limited sensitivity to conventional chemotherapy, radiochemotherapy, or immunotherapy alone [10–12], radiotherapy may sensitize immunotherapy and increase its efficacy. It was shown that radiotherapy enhanced the presentation of tumor-associated antigens, increased T-cell recognition and PD-L1 expression of tumor cells. The combination of radiotherapy with PD-1 blockade also increased endogenous T-cell infiltration of tumors and PD-L1 expression in tumor cells [35, 36]. It may be that the involvement of radiotherapy sensitized not only the local lesion, but also the abscopal metastatic lesion, which enhanced the efficacy of both radiotherapy and PD-1 blockade.

## Conclusions

Our cases highlighted the therapeutic potential of the combination of radiotherapy with immunotherapy for late-stage or recurrent ICC patients with low TMB, pMMR, MSS and negative PD-L1 expression, and expanded immunotherapy to those patients who were previously regarded as unsuitable for PD-1 blockade. This therapeutic efficacy may be applied not only to ICC, but also to other refractory cancers. Abscopal effects were also confirmed in our study with the combination, which enhanced the efficacy of both radiotherapy and immunotherapy with well tolerance and acceptable toxicity. Our study provided a new option to maximize the benefit for late-stage or refractory cancer patients in therapies involving PD-1 blockade.

## Data Availability

The datasets generated and/or analyzed during the current study are available from the corresponding author upon reasonable request.

## Abbreviations

CR: Complete response
DCR: Disease control rate
dMMR: Deficient mismatch repair
Gy: Gray
HBsAg: Hepatitis B surface antigen
HBV: Hepatitis B virus
ICC: Intrahepatic cholangiocarcinoma
INDEL: Insertion and deletion
MRI: Magnetic resonance imaging
MSI: Microsatellite instability
MSS: Microsatellite stable
NCCN: National comprehensive cancer network
NSCLC: Non-small cell lung cancer
ORR: Objective response rate
OS: Overall survival
PD-1: Programmed cell death protein 1
PD-L1: Programmed cell death ligand 1,
PET-CT: Positron emission tomography-computed tomography
PFS: Progression free survival
pMMR: Proficient mismatch repair
PR: Partial response
RT: Radiotherapy
SBRT: Stereotactic body radiotherapy
SD: Stable disease
TMB: Tumor mutation burden
WES: Whole-exome sequencing

## References

[1] Antwi SO, Mousa OY, Patel T (2018). Racial, ethnic, and age disparities in incidence and survival of intrahepatic cholangiocarcinoma in the United States; 1995-2014. Ann Hepatol.

[2] Shaib Y, El-Serag HB (2004). The epidemiology of cholangiocarcinoma. Semin Liver Dis.

[3] Banales JM, Cardinale V, Carpino G, Marzioni M, Andersen JB, Invernizzi P (2016). Expert consensus document: cholangiocarcinoma: current knowledge and future perspectives consensus statement from the European network for the study of cholangiocarcinoma (ENS-CCA). Nat Rev Gastroenterol Hepatol.

[4] Zhou M, Wang H, Zhu J, Chen W, Wang L, Liu S (2016). Cause-specific mortality for 240 causes in China during 1990-2013: a systematic subnational analysis for the global burden of disease study 2013. Lancet.

[5] Yamamoto M, Takasaki K, Yoshikawa T (1999). Lymph node metastasis in intrahepatic cholangiocarcinoma. Jpn J Clin Oncol.

[6] Lafaro KJ, Cosgrove D, Geschwind JF, Kamel I, Herman JM, Pawlik TM (2015). Multidisciplinary Care of Patients with intrahepatic cholangiocarcinoma: updates in management. Gastroenterol Res Pract.

[7] Cai JQ, Cai SW, Cong WM, Chen MS, Chen P, Chen XP (2014). Diagnosis and treatment of cholangiocarcinoma: a consensus from surgical specialists of China. J Huazhong Univ Sci Technolog Med Sci.

[8] Cai Y, Cheng N, Ye H, Li F, Song P, Tang W (2016). The current management of cholangiocarcinoma: a comparison of current guidelines. Biosci Trends.

[9] Weber SM, Ribero D, O'Reilly EM, Kokudo N, Miyazaki M, Pawlik TM (2015). Intrahepatic cholangiocarcinoma: expert consensus statement. HPB (Oxford).

[10] Tran Cao HS, Zhang Q, Sada YH (2018). The role of surgery and adjuvant therapy in lymph node-positive cancers of the gallbladder and intrahepatic bile ducts. Cancer.

[11] Squadroni M, Tondulli L, Gatta G, Mosconi S, Beretta G, Labianca R (2017). Cholangiocarcinoma. Crit Rev Oncol Hematol.

[12] Mavros MN, Economopoulos KP, Alexiou VG, Pawlik TM (2014). Treatment and prognosis for patients with intrahepatic cholangiocarcinoma: systematic review and meta-analysis. JAMA Surg.

[13] Rizvi NA, Hellmann MD, Brahmer JR, Juergens RA, Borghaei H, Gettinger S (2016). Nivolumab in combination with platinum-based doublet chemotherapy for first-line treatment of advanced non-small-cell lung Cancer. J Clin Oncol.

[14] Ott PA, Bang YJ, Berton-Rigaud D, Elez E, Pishvaian MJ, Rugo HS (2017). Safety and antitumor activity of Pembrolizumab in advanced programmed death ligand 1-positive endometrial Cancer: results from the KEYNOTE-028 study. J Clin Oncol.

[15] Overman MJ, Lonardi S, Wong KYM, Lenz HJ, Gelsomino F, Aglietta M (2018). Durable clinical benefit with Nivolumab plus Ipilimumab in DNA mismatch repair-deficient/microsatellite instability-high metastatic colorectal Cancer. J Clin Oncol.

[16] Le DT, Durham JN, Smith KN, Wang H, Bartlett BR, Aulakh LK (2017). Mismatch repair deficiency predicts response of solid tumors to PD-1 blockade. Science.

[17] Kim R, Kim D, Alese O, Li D, El-Rayes B, Shah N (2018). O-009 A Phase II multi institutional study of nivolumab in patients with advanced refractory biliary tract cancers (BTC). Ann Oncol.

[18] Arkenau HT, Martin-Liberal J, Calvo E, Penel N, Krebs MG, Herbst RS (2018). Ramucirumab plus Pembrolizumab in patients with previously treated advanced or metastatic biliary tract Cancer: nonrandomized, open-label, phase I trial (JVDF). Oncologist.

[19] Lin Jianzhen, Shi Weiwei, Zhao Songhui, Hu Jinwei, Hou Zheng, Yao Ming, Chrin Gungwei, Pan Jie, Hu Ke, Zhao Lin, Javle Milind, Wang Kai, Zhao Haitao (2018). Lenvatinib plus checkpoint inhibitors in patients (pts) with advanced intrahepatic cholangiocarcinoma (ICC): Preliminary data and correlation with next-generation sequencing. Journal of Clinical Oncology.

[20] Shen ZT, Zhou H, Li AM, Li B, Shen JS, Zhu XX (2017). Clinical outcomes and prognostic factors of stereotactic body radiation therapy for intrahepatic cholangiocarcinoma. Oncotarget.

[21] Mahadevan A, Dagoglu N, Mancias J, Raven K, Khwaja K, Tseng JF (2015). Stereotactic body radiotherapy (SBRT) for intrahepatic and hilar cholangiocarcinoma. J Cancer.

[22] Harada Y, Miyazaki S (2018). CyberKnife stereotactic radiosurgery for cholangiocarcinoma. Intern Med.

[23] Postow MA, Callahan MK, Barker CA, Yamada Y, Yuan J, Kitano S (2012). Immunologic correlates of the abscopal effect in a patient with melanoma. N Engl J Med.

[24] Koller KM, Mackley HB, Liu J, Wagner H, Talamo G, Schell TD (2017). Improved survival and complete response rates in patients with advanced melanoma treated with concurrent ipilimumab and radiotherapy versus ipilimumab alone. Cancer Biol Ther.

[25] Roger A, Finet A, Boru B, Beauchet A, Mazeron JJ, Otzmeguine Y (2018). Efficacy of combined hypo-fractionated radiotherapy and anti-PD-1 monotherapy in difficult-to-treat advanced melanoma patients. Oncoimmunology.

[26] Aboudaram A, Modesto A, Chaltiel L, Gomez-Roca C, Boulinguez S, Sibaud V (2017). Concurrent radiotherapy for patients with metastatic melanoma and receiving anti-programmed-death 1 therapy: a safe and effective combination. Melanoma Res.

[27] Chicas-Sett Rodolfo, Morales-Orue Ignacio, Castilla-Martinez Juan, Zafra-Martin Juan, Kannemann Andrea, Blanco Jesus, Lloret Marta, Lara Pedro C (2019). Stereotactic Ablative Radiotherapy Combined with Immune Checkpoint Inhibitors Reboots the Immune Response Assisted by Immunotherapy in Metastatic Lung Cancer: A Systematic Review. International Journal of Molecular Sciences.

[28] Fiorica F, Belluomini L, Stefanelli A, Santini A, Urbini B, Giorgi C, et al. Immune Checkpoint Inhibitor Nivolumab and Radiotherapy in Pretreated Lung Cancer Patients: Efficacy and Safety of Combination. Am J Clin Oncol 2018:1. 10.1097/COC.0000000000000428 29389733

[29] Sharabi A, Kim SS, Kato S, Sanders PD, Patel SP, Sanghvi P (2017). Exceptional response to Nivolumab and stereotactic body radiation therapy (SBRT) in neuroendocrine cervical carcinoma with high tumor mutational burden: management considerations from the center for personalized Cancer therapy at UC san Diego Moores Cancer center. Oncologist.

[30] Qin Q, Nan X, Miller T, Fisher R, Teh B, Pandita S (2018). Complete local and Abscopal responses from a combination of radiation and Nivolumab in refractory Hodgkin's lymphoma. Radiat Res.

[31] Formenti SC (2010). Immunological aspects of local radiotherapy: clinical relevance. Discov Med.

[32] Mou H, Yu L, Liao Q, Hou X, Wu Y, Cui Q (2018). Successful response to the combination of immunotherapy and chemotherapy in cholangiocarcinoma with high tumour mutational burden and PD-L1 expression: a case report. BMC Cancer.

[33] Sui M, Li Y, Wang H, Luo Y, Wan T, Wang X (2019). Two cases of intrahepatic cholangiocellular carcinoma with high insertion-deletion ratios that achieved a complete response following chemotherapy combined with PD-1 blockade. J Immunother Cancer.

[34] Luke JJ, Lemons JM, Karrison TG, Pitroda SP, Melotek JM, Zha Y (2018). Safety and clinical activity of Pembrolizumab and multisite stereotactic body radiotherapy in patients with advanced solid tumors. J Clin Oncol.

[35] Sharabi AB, Nirschl CJ, Kochel CM, Nirschl TR, Francica BJ, Velarde E (2015). Stereotactic radiation therapy augments antigen-specific PD-1-mediated antitumor immune responses via cross-presentation of tumor antigen. Cancer Immunol Res.

[36] Takamori S, Toyokawa G, Takada K, Shoji F, Okamoto T, Maehara Y (2018). Combination therapy of radiotherapy and anti-PD-1/PD-L1 treatment in non-small-cell lung Cancer: a mini-review. Clin Lung Cancer.

